# Spectrum of *MYO7A* Mutations in an Indigenous South African Population Further Elucidates the Nonsyndromic Autosomal Recessive Phenotype of DFNB2 to Include Both Homozygous and Compound Heterozygous Mutations

**DOI:** 10.3390/genes12020274

**Published:** 2021-02-15

**Authors:** Rosemary Ida Kabahuma, Wolf-Dieter Schubert, Christiaan Labuschagne, Denise Yan, Susan Halloran Blanton, Michael Sean Pepper, Xue Zhong Liu

**Affiliations:** 1 Department of Otorhinolaryngology, University of Pretoria, Pretoria 0001, South Africa; 2 Departments of Biochemistry, Genetics and Microbiology, Faculty of Natural and Agricultural Sciences, University of Pretoria, Pretoria 0001, South Africa; wolf-dieter.schubert@up.ac.za; 3 Inqaba Biotechnical Industries, Pretoria 0002, South Africa; Christiaan.Labuschagne@inqababiotec.co.za; 4 Department Otolaryngology, University of Miami Miller School of Medicine, Miami, FL 33136, USA; DYan@med.miami.edu (D.Y.); SBlanton@med.miami.edu (S.H.B.); 5 Dr. John T. Macdonald Foundation Department of Human Genetics, and John P. Hussman Institute for Human Genomics, University of Miami Miller School of Medicine, Miami, FL 33136, USA; 6 Department Immunology and SAMRC Extramural Unit for Stem Cell Research and Therapy, Faculty of Health Sciences, Institute for Cellular and Molecular Medicine, University of Pretoria, Pretoria 0001, South Africa; Michael.Pepper@up.ac.za

**Keywords:** DFNB2, *MYO7A* gene, recessive hearing loss, spectrum of *MYO7A* mutations, South African, homozygous, compound heterozygous, sub-Saharan Africa

## Abstract

*MYO7A* gene encodes unconventional myosin VIIA, which, when mutated, causes a phenotypic spectrum ranging from recessive hearing loss DFNB2 to deaf-blindness, Usher Type 1B (USH1B). *MYO7A* mutations are reported in nine DFNB2 families to date, none from sub-Saharan Africa.In DNA, from a cohort of 94 individuals representing 92 families from the Limpopo province of South Africa, eight *MYO7A* variations were detected among 10 individuals. Family studies identified homozygous and compound heterozygous mutations in 17 individuals out of 32 available family members. Four mutations were novel, p.Gly329Asp, p.Arg373His, p.Tyr1780Ser, and p.Pro2126Leufs*5. Two variations, p.Ser617Pro and p.Thr381Met, previously listed as of uncertain significance (ClinVar), were confirmed to be pathogenic. The identified mutations are predicted to interfere with the conformational properties of myosin VIIA through interruption or abrogation of multiple interactions between the mutant and neighbouring residues. Specifically, p.Pro2126Leufs*5, is predicted to abolish the critical site for the interactions between the tail and the motor domain essential for the autoregulation, leaving a non-functional, unregulated protein that causes hearing loss. We have identified *MYO7A* as a possible key deafness gene among indigenous sub-Saharan Africans. The spectrum of *MYO7A* mutations in this South African population points to DFNB2 as a specific entity that may occur in a homozygous or in a compound heterozygous state.

## 1. Introduction

*MYO7A* encodes an unconventional myosin, myosin VIIA, which, when mutated, causes a phenotypic spectrum ranging from recessive nonsyndromic hearing loss (DFNB2) to syndromic deaf-blindness, Usher Type 1B (USH1B). Studies in transfected mouse hair cells demonstrated that DNFB2 mutant myosin VIIA protein localizes correctly to the hair cell stereocilia, yet the USH1B myosin VIIA proteins do not, confirming that, in contrast to USH1B-associated alleles, the DNFB2-associated alleles retain some residual function and that DFNB2 and USH1B are different ends of the same disease spectrum. Furthermore, elucidation of the three distinct forms of Usher Syndrome Type 1 demonstrated that USH1B, USH1C, and USH1D are distinct genetic disorders caused by mutations in three different genes, *MYO7A*, *CHD23*, and *USH1C*, respectively [1,2,3,4,5]. DFNB2 is a recessive form of prelingual sensorineural hearing loss that may, on rare occasions, occur with vestibular dysfunction and vertigo.

### 1.1. Background Information

Tip links and the mechanoelectrical transduction apparatus of the cochlea hair cells.

The hearing mechanism at the cochlear level depends on the integrity of both the physiologic conditions and the mechanical conditions in the cochlea. Of these factors, the most vital is the state of the hair cells and their stereocilia. Inner ear hair cell stereocilia exhibit links between the tips and sides of adjacent stereocilia [6,7,8]. These links are part of the mechanoelectrical transduction (MET) apparatus of the sensory cells of the inner ear [9,10,11]. The links also ensure that the stereocilia are mechanically integrated into bundles that can withstand the repeated stress caused by shearing movements that occur during sound conduction [6,7,8,12].

**Figure 1:**
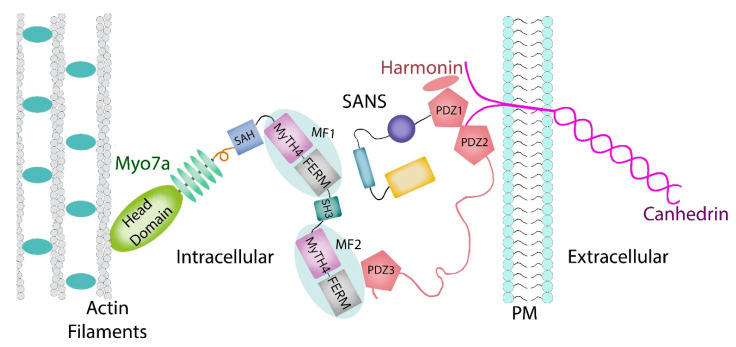
Assembly of the myosin 7a-SANS-harmonin complex at a cellular level.

Myosin VIIA belongs to the myosin superfamily, a large and diverse family of over twenty proteins whose members are involved in a number of cellular pathways [13,14]. In humans, myosins are grouped into twelve classes. Myosin sub-class VIIA is expressed in the inner ear. Specifically, myosin VIIA is a vital component of the tripartite complex in the hair cell stereocilia where it helps to maintain the mechanical tension across the cadherin links [12,15,16] and to transport the protein complex to the tips [17,18,19]. Myosin VIIA forms tripartite complexes involving two modular adaptor proteins, SANS and Harmonin, that anchor the cytoplasmic domains of cadherins to the actin cytoskeleton [12,17,20,21]. Both harmonin and myosin VIIA localize along the length of stereocilia, but the three Usher proteins are localized at the upper-tip link density (UTLD), where they associate with the tip link and regulate the function of MET [22,23,24,25]. In the assembly (Figure 1), the cytoplasmic tail of CDH23 binds to the PDZ domain scaffolding protein harmonin (USH1C), which in turn forms a tripartite complex with the ankyrin-repeat *SANS* adaptor (USH1G), and which in turn binds to the tail domain of myosin VIIA [17,26]. The cooperation of these three proteins is essential because the earliest connections between the growing stereocilia are critical for shaping the hair bundle as a coherent unit. Failure of this interaction leads to disorganized stereocilia and to hearing loss.

### 1.2. Myosin VIIA Heavy Chain Structure and Function

Structurally (Figure 1), the well conserved myosin VIIA heavy chain contains domains that provide for its motor-and cargo-binding functions [27,28,29]. The head and neck are composed of a conserved N-terminal region containing sixty amino acids, a characteristic motor domain, and five isoleucine-glutamine (IQ) motifs that bind calmodulin [30,31] and/or specific light chains. Next is a stable single α–helix (SAH) [29] followed by a tandem repeat of myosin tail homology 4 (MyTH4) domains and partial four-point 1, ezrin, radixin, and moesin (FERM) domains that are separated by an SH3 subdomain. Myosin VIIA demonstrates actin activated ATPase activity [32,33].

The highly conserved motor domain contains ATP- and actin binding sites associated with nucleotide binding, hydrolysis and product release, essential for myosin enzyme motility, while the tail region mediates dimerization and binding to other proteins or cargo [27]. Furthermore, myosin VIIA motor activity plays a key role in the differentiation and organization of hair cell stereocilia [34]. The myosin MF domains bind to the cytoplasmic tails of signaling receptors and adhesion receptors and interact with adapter proteins [27,35,36,37,38] as well as play a major role in autoinhibition [39]. Studies across species show that FERM subdomains exhibit class-specific sequence variations yet retain a very high degree of conservation of the supramodule, and of the cloverleaf configuration which controls not only the three lobes of the FERM domain, but the rotational freedom of the MyTH4 as well. Indeed, though the FERM domain may bind to actin binding proteins, signaling receptors and adhesion molecules [35,36,37,38,39,40], it remains vital in the autoinhibition of mammalian Myo7a and Myo10 as well as in Drosophila Myo7a [28,39,40,41].

Biochemical experiments have shown that myosin VIIA has a compact structure that exhibits, in its monomeric form, intramolecular bending as well as a unique regulatory mechanism [39]. Furthermore, myosin VIIA adopts a bent conformation in the presence of ATP and is extended in the absence of ATP [39]. A portion of the tail interacts with the motor domain [39] in the bent conformation. Yang et al., 2009 [39] demonstrated that, upon release of the tail inhibition, the full length myosin VIIA can transport its cargo molecules, such as the USH1 proteins. Myosin VIIA has been shown to associate with large protein complexes, either transporting them to a particular site or stabilizing the cargo in a specific position [27,42]. In the neuroretina, myosin VIIA plays a key role in the distribution and migration of retinal pigment epithelial (RPE) melanosomes and phagosomes [43,44], and enables the regulation of opsin transport in retinal photoreceptors [45,46].

### 1.3. MYO7A Gene Expression and Phenotypes

*MYO7A* is expressed in cochlea neuroepithelia, vestibular neuroepithelia, retinal photoreceptor cells, and retinal pigment epithelium. In humans, *MYO7A* is expressed in both the pigment cells and in the photoreceptor cells in the retina. In mice, however, myo7a is not expressed in the photoreceptor cells but only in pigment epithelium. For this reason, the shaker-1 mouse has no retinal defects, only demonstrating hearing and balance disorders [47]. In humans, a primary rod and cone defect were shown to cause the syndrome depicting the retinal defects associated with the vestibuloauditory defects of USH1B [47]. The DFNB2 mutations do not give rise to retinal defects and blindness, even though they are found in the same gene as the USH1B mutations.

Non-syndromic sensorineural hearing loss was reported in 22 individuals from a large consanguineous family and mapped to chromosome 11q13 [48] through linkage analysis. It was subsequently identified to be due to homozygous mutations in the *MYO7A* gene [49]. Following on these findings, two Chinese families were identified with homozygous and heterozygous *MYO7A* mutations [50], extending the DFNB2 genotype from homozygous recessive mutations alone to include compound heterozygous mutations. Since then, characterization studies point to DFNB2 and Usher syndrome as representing a disease spectrum with variable expressivity. Molecular findings demonstrate that the DNFB2-associated alleles retain some residual function at the hair cell apices in contrast to USH1B-associated alleles [51].

Confirmation of DFNB2 as a separate entity has been relatively slow, mainly because, initially, families from other population groups could not be identified. These negative findings led to calling into question the existence of DFNB2 altogether by the researchers who felt there was disparity in the ‘alleged causative mutations’ and the nonsyndromic phenotype [52]. Prior to the current study findings (Table 1), no DFNB2 families had been reported from sub-Saharan Africa. Over time, DFNB2 linked *MYO7A* mutations have now been identified in different population groups including Pakistani [19], Iranian [53], Middle Eastern populations including Jewish and Arab Palestinian populations [54], and Chinese [50,55,56] (Table 2). Mutations have been reported mainly among populations practicing close family marriages. However, DFNB2 has been suspected, either through linkage studies, or through characterization studies of *MYO7A* mutations, in a wider spectrum of population groups. These include Tunisian, Pakistani, Saudi Arabian, Iranian, Chinese, Japanese, Swedish, Spanish, Palestinian, and Moroccan population groups respectively [19,50,53,55,56,57,58,59,60,61]. Out of these, clearly characterized mutations in *MYO7A* have been confirmed in fourteen DFNB2 families to date (Table 2). We now report our findings demonstrating the existence of DFNB2, due to both homozygous and compound heterozygous *MYO7A* mutations, among sub-Saharan African populations from South Africa.

## 2. Materials and Methods

This study was approved by the Ethics Committee of the Faculty of Health Sciences, University of Pretoria, South Africa, Ethics Approval number 395/2014, and the Institutional Review Board of the University of Miami, Miller School of Medicine, Miami Fl, USA. A signed informed-consent form was obtained from each participant or, in the case of a minor, from the parents.

### 2.1. Subjects

We included in this study 94 GJB2 mutation-negative indigenous sub-Saharan African individuals representing 92 families from the Limpopo Province of South Africa. Of these, 23 individuals had a definite family history of hearing loss, 49 individuals had none and 22 individuals were uncertain. Since a three-generation pedigree was not available in some cases, we did not group multiplex families according to an inheritance pattern.

### 2.2. Clinical Evaluation

Clinical evaluation included a thorough physical examination and otoscopy in all cases. Vestibular assessment was performed according to the “The ten-minute examination of the dizzy patient” protocol [62]. Additional evaluations, including a high-resolution, thin-section computed tomography (CT) and magnetic resonance imaging (MRI) of the temporal bone, were performed when possible. DNA was extracted from peripheral blood leukocytes of probands according to the standard procedures. SNHL was established via the standard audiometry in a soundproofed room according to the current clinical standards. HL was congenital onset or prelingual onset with a severity ranging from severe to profound. Assessment for retinopathy was performed by ophthalmologists at the local hospitals where they were referred to at least three occasions for each individual. Fundoscopy as well as tests for visual acuity and visual fields were done during the assessments. ERG and fundus photos were not performed due to lack of the equipment at the local hospitals. The initial evaluation was done in childhood at school entry during middle school. The third evaluation was done more recently at the time of family recruitment for the current study. The clinical records obtained at all these assessments together with history taken during vestibular assessment were used to determine the presence or absence of visual defects.

### 2.3. Sequencing

Using the Agilent SureDesign online tool (Available online: https://earray.chem.agilent.com/suredesign/ (accessed on 23 November 2020)), a SureSelect custom kit (Agilent, Santa Clara, CA, USA) was designed to include all exons, 5′ UTRs and 3′ UTRs of 180 known and candidate deafness causing genes (Supplementary Table S1) [63]. This custom capture panel (MiamiOtoGenes), with a target size of approximately 1.158 MB encompassing 3494 regions, covers genes associated with both syndromic and non-syndromic forms of HL. The targeted sequencing was processed at the Hussman Institute for Human Genomics (HIHG) Sequencing core, University of Miami. The Agilent’s SureSelect Target Enrichment (Agilent, Santa Clara, CA, USA) of coding exons and flanking intronic sequences in-solution hybridization capture system was used following the manufacturer’s standard protocol. Adapter sequences for the Illumina HiSeq 2000 were ligated, and the enriched DNA samples were prepared using the standard methods for the HiSeq 2000 instrument (Illumina). Through the sample preparation, average insert size was 180 bp and paired end reads were used. Regions with lower coverage were not subjected to additional sequencing.

### 2.4. Bioinformatics Analysis

The Illumina CASAVA v1.8 pipeline was used to assemble 99 bp sequence reads. Burrows–Wheeler Aligner (BWA) was applied for alignment of sequence reads to the human reference genome (hg19) [64], and variants were called using FreeBayes [65]. Genesis 2.0 (Available online: https://www.genesis-app.com/ (accessed on 23 November 2020)) was then used for variant filtering based on quality/score read depth and minor allele frequency (MAF thresholds of 0.005 for ARNSHL and 0.0005 for ADNSHL variants) as reported in dbSNP141, the National Heart, Lung, and Blood Institute Exome Sequencing Project Exome Variant Server, Seattle, WA Project (Exome Variant Server 2012), Exome Aggregation Consortium (ExAC) browser (Available online: http://exac.broadinstitute.org/ (accessed on 23 November 2020)), gnomAD, the 1000 Genome Project Database, and our internal database of >3000 samples from European, Asian, and American ancestries.

Variants meeting these criteria were further annotated based on their presence and pathogenicity information in Human Gene Mutation Database (HGMD; Available online: http://www.hgmd.cf.ac.uk (accessed on 23 November 2020)), the Deafness Variation Database (DVD) (Available online: deafnessvariationdatabase.org (accessed on 23 November 2020)), and ClinVar (Available online: http://www.ncbi.nlm.nih.gov/clinvar/ (accessed on 23 November 2020)). In the final step, all variants were re-classified based on the American College of Medical Genetics and Genomics (ACMG) and Association for Molecular Pathology (AMP) guidelines [66], together with the variant interpretation guidelines for genetic hearing loss as published by Oza et al., 2018 [67]. These guidelines recommend the use of specific standard terminology for DNA variants in five categories to include pathogenic, likely pathogenic, uncertain significance, likely benign, and benign. They describe criteria using evidence from population data, computational data, functional data, and segregation data for variant interpretation. Copy number variation (CNV) calling was performed using an R-based tool [68]. This method normalizes read-depth data by sample batch and compares median read-depth ratios using a sliding-window approach.

Sanger sequencing was used for the confirmation of variant calls and PCR for the CNVs. Family members, when available, were used for segregation, de novo status, and trans configuration of biallelic variants. During the interpretation, we also considered phenotypic correlations between the gene variants and their reported phenotypes.

In silico, 3D modelling was performed to investigate and predict the possible effects of the mutations on the protein product.

## 3. Results

### 3.1. Family Pedigrees

From a previous study involving 184 congenitally deaf indigenous African individuals from the Limpopo province of South Africa, all negative for *GJB2* mutations, the *GJB6*-D13S1830 deletion and four mitochondrial mutations, A1555G, A3243G, A7511C and A7445G [69], a cohort of 94 individuals representing 92 families was assessed for mutations in 180 deafness genes by capture targeted massively parallel sequencing (CTMPS) using the MiamiOtogene panel [70].

Eight MYO7A variants were detected in ten individuals (including two sibling pairs) from this cohort. All were adults between 26 and 37 years of age.

Two new families with deaf family members identified in the course of family tracing agreed to be included in the study and were recruited. These families are branches of Family TS074/TS093, and are designated as Branch B and Branch C, respectively, on the pedigree charts.

**Figure 2:**
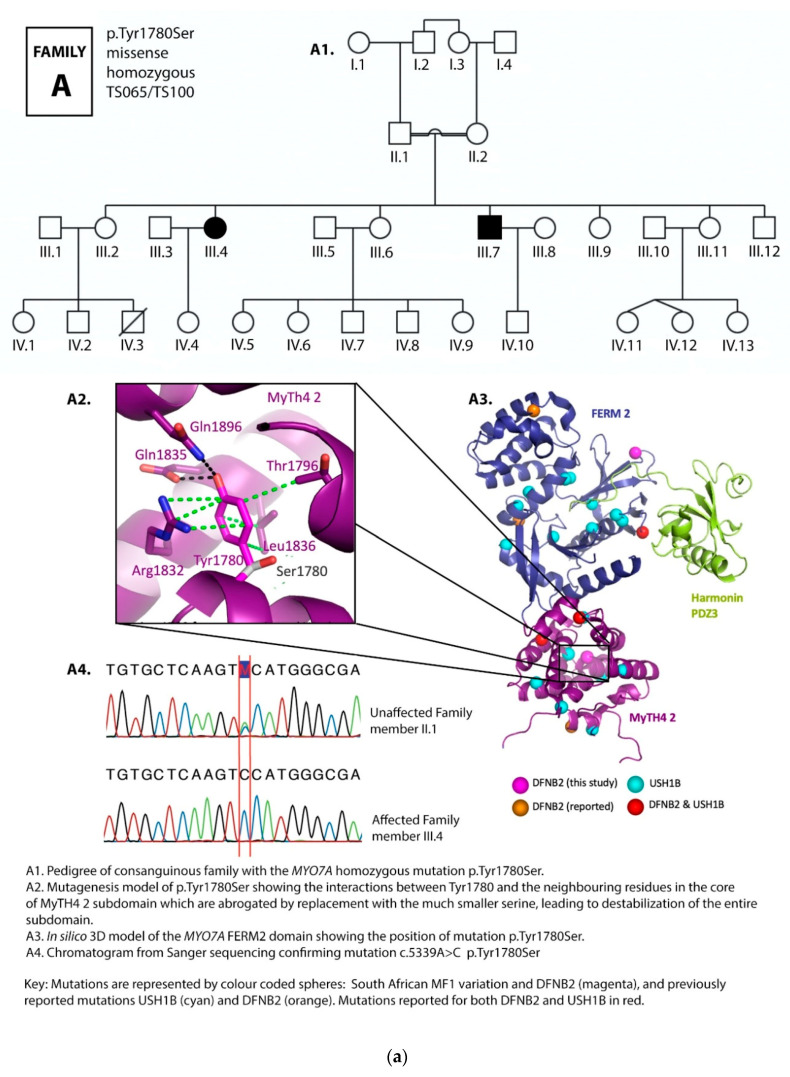

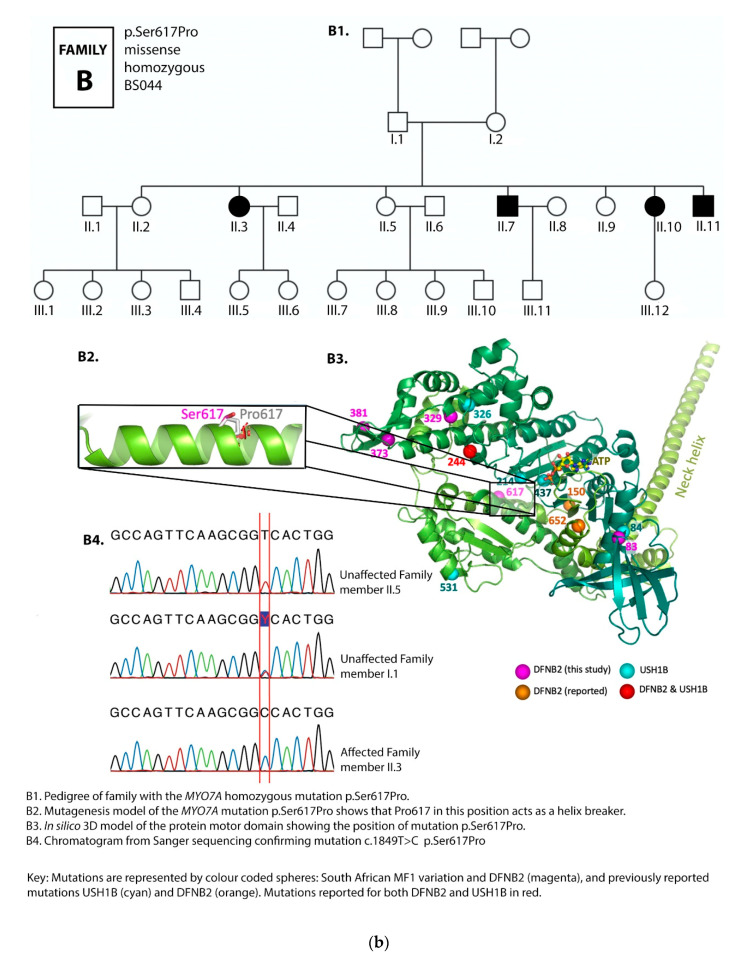

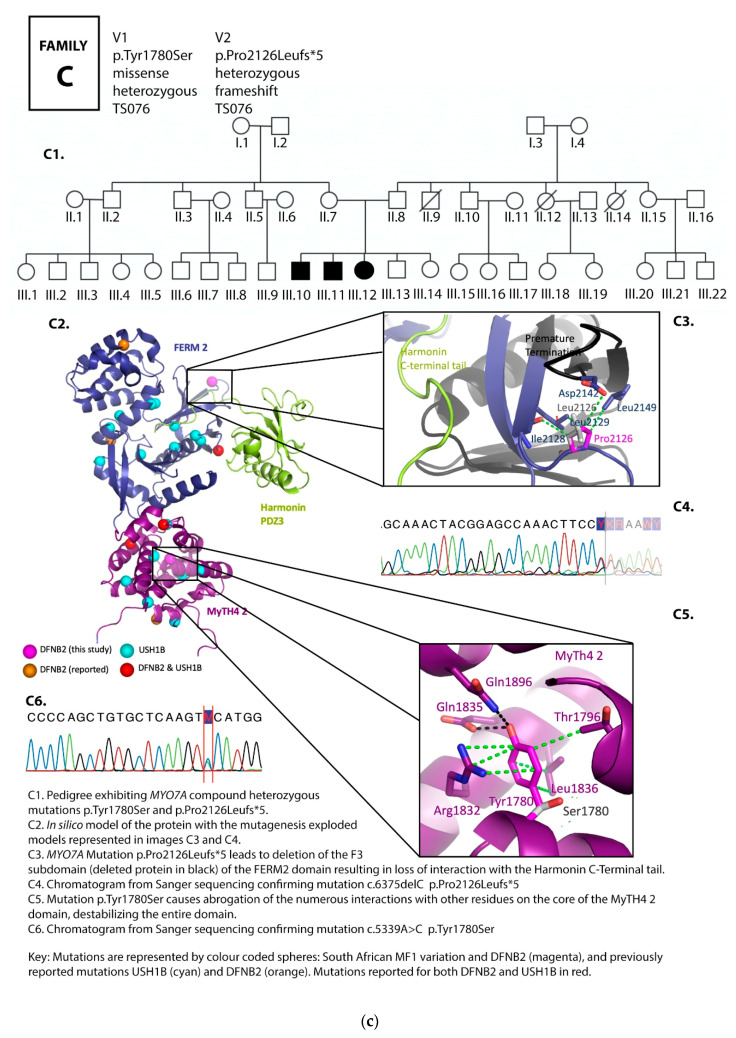

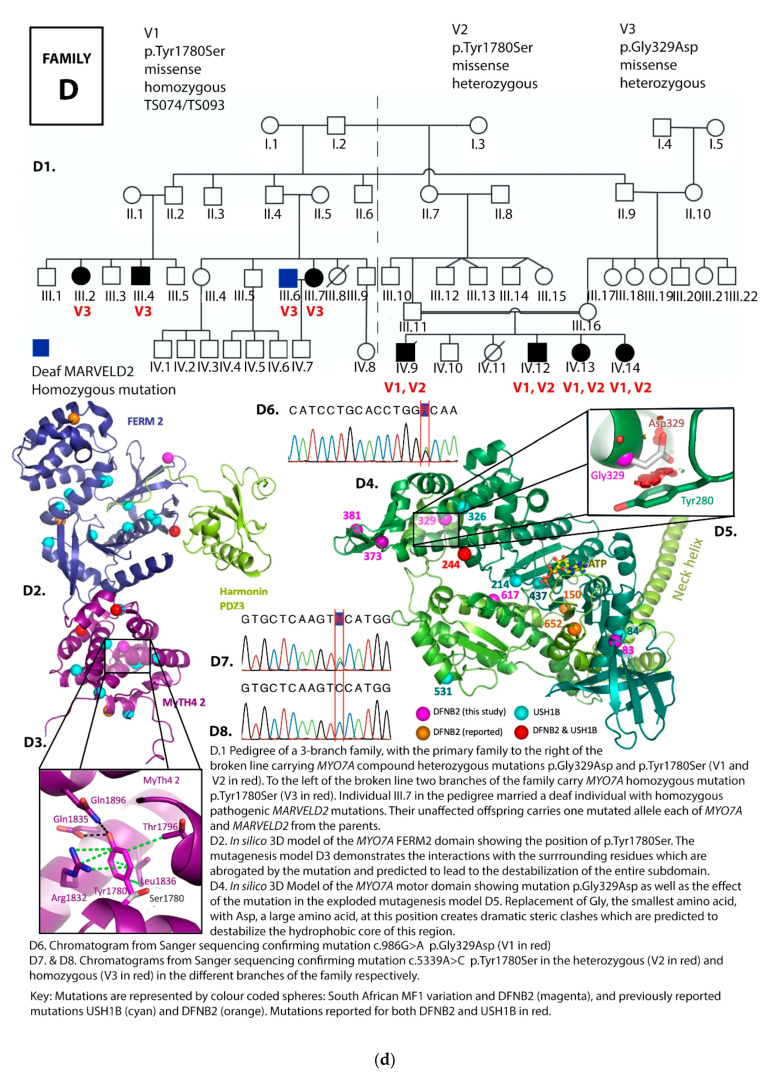

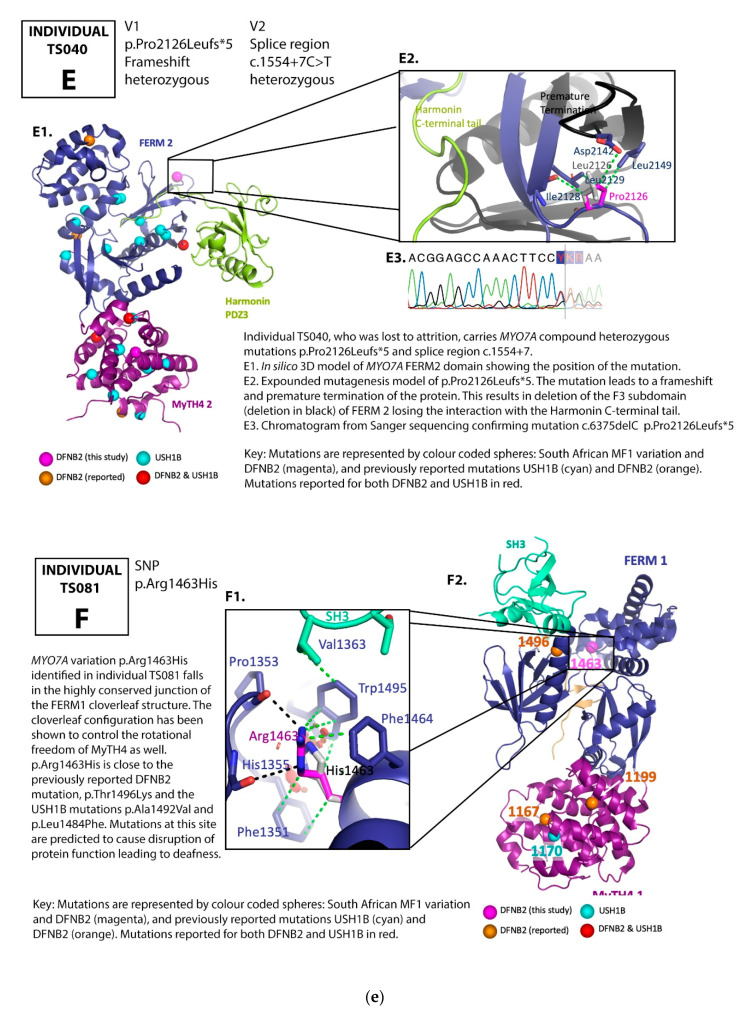

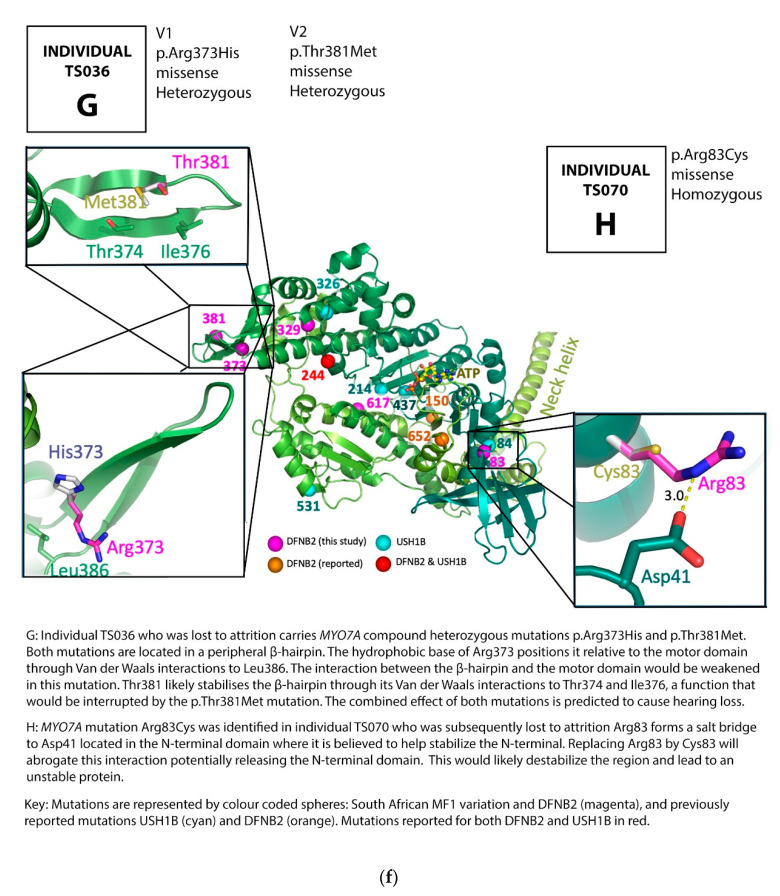
(**a**) Pedigree and mutation profile of DFNB2 in Family A; (**b**) pedigree and mutation profile of DFNB2 in Family B; (**c**) pedigree and mutation profile of DFNB2 in Family C; (**d**) pedigree and mutation profile of DFNB2 in Family D; (**e**) mutation profile and variant of DFNB2 in individuals TS040 and TS081; (**f**) mutation profiles of DFNB2 in individuals TS036 and TS070.

All families demonstrated co-segregation of the MYO7A mutations with hearing loss. (Figure 2, Table 1 and Table 2).

### 3.2. MYO7A Mutations

The eight *MYO7A* variants, including a splice site variant, identified in the South African cohort were ascertained among the respective family members. Out of thirty-two family members assessed, variants presenting both in the homozygous and in the compound heterozygous states were identified among seventeen adult individuals. All seventeen affected individuals demonstrated bilateral profound sensorineural hearing loss but no visual defects. One deaf individual, a 58-year old female, exhibited a subjective mild vestibular defect. Although she was not available for vestibular assessment, we were able to interview her via telephone. She, however, had normal vision and visual fields according to her medical records. All five available families demonstrated perfect co-segregation between the variants and the phenotype, with only the individuals carrying homozygous or compound heterozygous states exhibiting nonsyndromic hearing loss.

**Figure 3:**
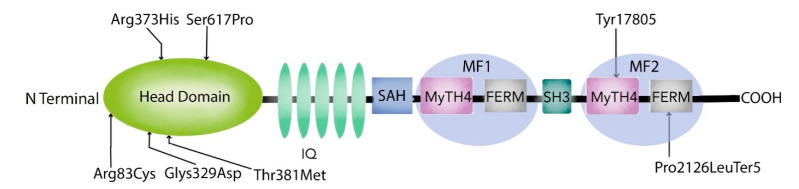
Diagrammatic representation of Myosin 7A indicating the mutations in DFNB2 South African families.

Four novel mutations, p.Gly329Asp, p.Arg373His, p.Tyr1780Ser, and p.Pro2126Leufs*5, as well as reported mutations p.Ser617Pro and p.Thr381Met, were identified (Table 1 and Table 2; Figure 2a–f, Figure 3).

Mutation p.Tyr1780Ser was the most common variant, found in both homozygous and compound heterozygous states in four families. Three families carried the p.Tyr1780Ser mutation in the homozygous state, while it was presented in the compound heterozygous state in one family. In families D1–D3, it was found in both the homozygous and compound heterozygous genotypes among different branches of the family (Table 1, Figure 2d). The spectrum of mutations identified in the current study were compared to known DFNB2 and USH1B mutations (Table 3). These results are further referred to in Section 4.

## 4. Discussion

Current understanding, based on years of the cumulative research on myosin VIIA, reveals that, in the inner ear, defective myosin VIIA will affect not only the morphology and function of hair cell stereocilia, but the whole MET process of the hair cells, leading to hearing loss. Studies have demonstrated (as confirmed by electron microscopy) that the wild type, full-length human myosin VIIA is a monomer that demonstrates tail-dependent inhibition, and that, in its compact structure, the protein tail domain is bent back towards the head-neck domain [28,39]. Furthermore, at high ionic strength and in the presence of Ca^2+^, the molecule is extended. The researchers also concluded that the release of this tail-dependent inhibition enables the wild type full-length human myosin VIIA to transport its cargo molecules, such as USH1 proteins. Current understanding also states that, though DNFB2-associated alleles retain some residual function in localizing accurately to the hair cell stereocilia, the mutant unconventional myosin VIIA proteins are unable to support normal hearing function [19].

In silico 3D-modelling in the current study demonstrated the interruption or abrogation of multiple interactions between the mutant and neighbouring residues.

The mutations in the myosin VIIA motor domain identified in our study, p.Arg83Cys, p.Gly329Asp, p.Arg373His, p.Thr381Met, p.Ser617Pro, are predicted to interfere with the conformational properties of the protein through the disruption of the numerous ionic interactions between the mutated and the neighbouring residues (Figure 2b,d,f and Figure 3). These effects could cause hearing loss through any one of the following mechanisms: functional disruption of the movement of the myosin VIIA motor head, the protein’s cargo-carrying function, as well as the formation and function of the protein complexes at the MET apparatus.

Though the function of the N-terminal domain is currently unknown, arginine 83, a positively charged amino acid, forms a salt bridge to aspartate 41 (Asp41) located in the N-terminal domain, and probably stabilizes the N-terminal. Replacing Arginine 83 by a cysteine (a neutral amino acid) will abrogate this interaction potentially releasing the N-terminal domain (Figure 2f) and, in so doing, destabilizing the region and leading to an unstable protein, and, we predict, through this, causing hearing loss.

Glycine is the smallest amino acid with a side chain consisting of hydrogen while aspartate is a large amino acid. Glycine 329 closely packs on to tyrosine 280. Replacing Glycine 329 by aspartate (Asp329) will create dramatic steric clashes (Figure 2d) which will destabilize the whole hydrophobic core of the region and affect the folding of the protein. The function of the entire protein will likely be abolished.

Arginine 373 is located in a peripheral β-hairpin (Figure 2f). Its hydrophobic base positions the β-hairpin relative to the motor domain through van der Waals interactions to leucine 386. Replacing arginine 373 by histidine (Arg373His) will weaken the interaction between the β-hairpin and the motor domain. Although the function of the β-hairpin is unknown, threonine 381 is located in the same peripheral β-hairpin as arginine 373, where it stabilizes the β-hairpin by van der Waals interactions to threonine 374 and isoleucine 376. Replacing threonine 381 by methionine may affect the positioning and function of the β-hairpin. In the current study, one of the families carries both β-hairpin mutations Arg373His and Thr381Met in the compound heterozygous state, which, from our 3D model results, we predict to be pathological when combined together at these sites (Figure 2f, Table 1 and Supplementary Table S1).

Serine 617 is located in the middle of an α-helix. Replacing serine 617 by proline—a known “helix breaker”—will cause a kink or bend in the α-helix, interfering with its folding and the conformation of the protein, which will result in defective protein function (Figure 2b). This, in turn, would lead to hearing loss. The family with this mutation exhibited segregation of hearing loss with the homozygous genotype (Figure 2b), demonstrating the severe effect of the mutation as a helix breaker.

The two mutations in the C-terminal MyTH4-FERM domain, p.Tyr1780Ser and p.Pro2126Leufs*5, both show interesting characteristics on 3D modelling.

Tyrosin 1780 extensively interacts with numerous other side chains through van der Waals interactions, hydrogen bonds, as well as π π-stacking interactions. Replacing it with serine (Ser1780) leaves the pocket in this subdomain empty (Figure 2a,c,d). The effect is a transformation of the entire domain leading to severe disruption of the conformational properties of the protein, and we predict, through this, to hearing loss. In the large pedigree with three affected branches of the family, this mutation occurred both in the homozygous and compound heterozygous states, segregating clearly with deafness in all cases.

Proline 2126 (Pro2126) is located in the second MyTH7 [89], the last subdomain of the FERM 2 domain (Figure 2c,e). The mutation p.Pro2126Leufs*5 leads to destabilization of this domain in two ways. First, it leads to a frameshift and premature termination of the myosin VIIA protein five amino acids into the new reading frame (Figure 2c,e). This abolishes the critical site for the interaction between the tail and the motor domain, a site essential for the autoregulation of the protein and critical for protein function [39]. Secondly, the mutation is close to the harmonin PDZ3 recognition and binding site. Pro2126Leufs*5 deletes the F3 lobe of FERM 2 (deletion in black, Figure 2c,e), losing the interaction with the Harmonin C-terminal tail.

Mutations in the second MyTH4-FERM (MF2) domain, p.Tyr1780Ser and p.Pro2126Leufs*5, occurred in more than one family in this South African cohort (Table 1, Supplementary Table S1).

Following the original alignments by Chen et al., 1996 [3], subsequent studies demonstrated that the crystal structures of FERM domains consist of three lobes (subdomains) organized in a cloverleaf pattern, each containing 100 amino acids [27,90,91,92]. The sequences immediately following the first two subdomains are highly conserved among myosin VIIAs, myosin VII, and myosin VIIBs, and are as conserved as or more highly conserved than the two lobes of the FERM domain that directly precede them (Supplementary Table S2). Due to this, the 100-amino-acid stretch following FERM 1 and the 75-amino-acid stretch following FERM 2 domains have been referred to as the first and second MyTH7 domains, respectively.

Characterization studies (Supplementary Table S2) on the second MyTH7 subdomain of myosin 7a confirmed important mechanisms of action in its role [39,89]. These studies demonstrated that it is the second FERM domain in the myosin 7a tail that binds with actin filaments, and, through cosedimentation experiments, the researchers further revealed that the second FERM domain bound to actin in a saturable manner. They also demonstrated that truncated and unregulated myosin 7a-TD3, in which the C-terminal FERM/MyTH7 domain is missing, was extended with a single clearly discernible motor domain strongly angled to the rest of the molecule, confirming that the MyTH7 subdomain is required to effect the folded conformation [39,89]. Moreover, through MgATPase assays, the conserved SH3-binding motif in the MyTH7 subdomain was shown not to be involved in the regulation of myosin 7a. Finally, when the highly conserved residues Arg2140 and Lys2143, located in the second MyTH7 subdomain, were mutated through point mutations to alanines (Ala2140 and Ala2143 respectively), the mutant remained extended and resembled the myosin 7a-TD3 construct, even in the presence of ATP at low ionic strength, demonstrating a loss of autoregulation. This structure was confirmed by electron microscopy [39,89]. Together, these findings confirmed that it is the second MyTH7 subdomain that is essential for the conformation and the regulation of the protein.

The MYO7A C-terminal MyTH4-FERM domain (MF2) recognizes harmonin through the extended PDZ3 domain (Figure 1) of USH1C (harmornin). Several reported Myo7a deafness mutants that mapped to the surface of MF2 were shown to disrupt harmonin binding, revealing the molecular basis for the disruption of the tripartite complex assembly and of mechanotransduction [27,29,93].

Proline 2126 (Pro2126) is located in the second MyTH7 [89], the last subdomain of the FERM 2 domain (Figure 2c,e). As mentioned above, this subdomain has been shown to be critical for the interaction with the Harmonin PDZ3 domain [29,93]. The C-terminus of Harmonin extends into a deep pocket created by the myosin VIIA FERM 2 domain. Yang et al., 2009 [39] found that point mutations in the MyTH7 subdomain carried a similar enzymatic signature as larger truncations, including ones that even deleted all of the tail. Furthermore, evidence from the reported human truncating mutation in myosin VIIA linked to USH1B [94] that results in a myosin molecule similar in length to the TD3 construct reported by Yang et al., 2009 [39] leads us to predict that our mutant too is unregulated (Supplementary Table S2). The combined effect of the p.Pro2126Leufs*5 mutation identified in South African DFNB2 families on the myosin molecule would likely lead to a non-functional, unregulated protein that fails to form functional tripartite complexes in the hair cell stereocilia and the tip-links, leading to the failure of the MET process, and, we predict, through this, result in hearing loss.

### DFNB2 versus Usher 1B Syndrome

Wu et al., 2011 [27] mapped and analyzed 17 missense mutations (non-truncating or deletion mutations were excluded) reported in Usher syndrome onto a crystal structure of myosin VIIA MyTH4-FERM domains. They noted that 1 was in the SH3, 9 were located in FERM, while 7 were located in MyTH4 domains. They further subdivided the mutations into classes depending on location. They predicted that the mutations in the folding core of MyTH4 and FERM domains (Class 1) negatively affected the folding of the MyTH4-FERM supramodule, while mutations in the second class would likely affect interactions between domains or interactions among the tripartite protein complexes, such as between myosin VIIA and Sans. They demonstrated that Glu1349, which is closely involved in myosin VIIA binding to Sans CEN1, when mutated to Lys1349, weakened the binding by 20-fold [27]. The third mutation class involved the conserved solvent exposed residues in F1 and F3. These were predicted to interfere with interactions of the protein with other molecules. The fourth mutation class consisted of residues outside the prediction of the MFS-CEN structure. These were analyzed through sequence analysis. They found a conserved R-X-X-X-P-X-(X)-X-E motif in all MyTH4 domains in loops a9/a10 and a10. Furthermore, they noted that the orientation of a9 and a10 was determined by the salt bridges between the Arg and Glu residues, while the turn structure specifically of the a9/a10 loop was facilitated by the Pro residue (Pro1244). They therefore predicted that, with regard to myosin VIIA MyTH4-FERM tandems in humans, mutations in Arg or Pro in this particular motif would cause deaf-blindness.

**Figure 4:**
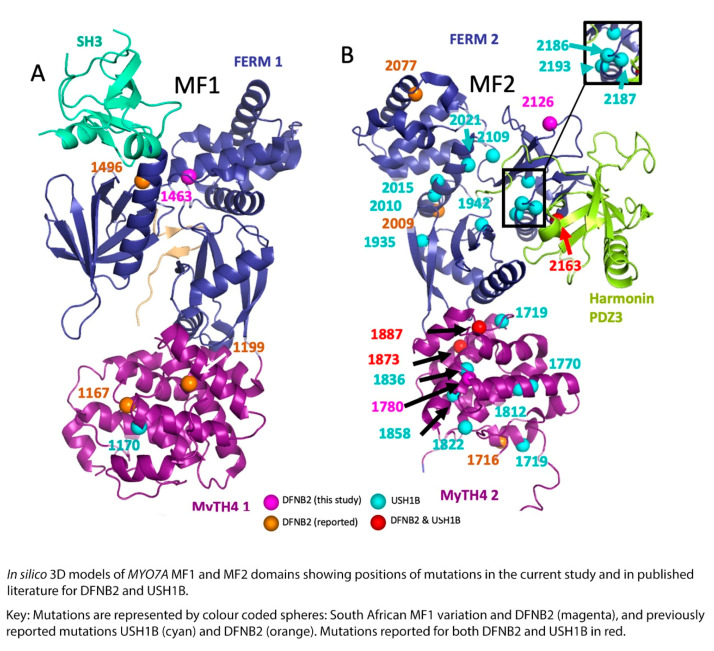
Mapping of DFNB2 and USH1B mutations from the current study and published literature located in the *MYO7A* gene MF domains. Subfigure **A** represents MyTH4-FERM 1 domain (MF1) and subfigure **B** represents MyTH4-FERM 2 domain (MF2).

**Figure 5:**
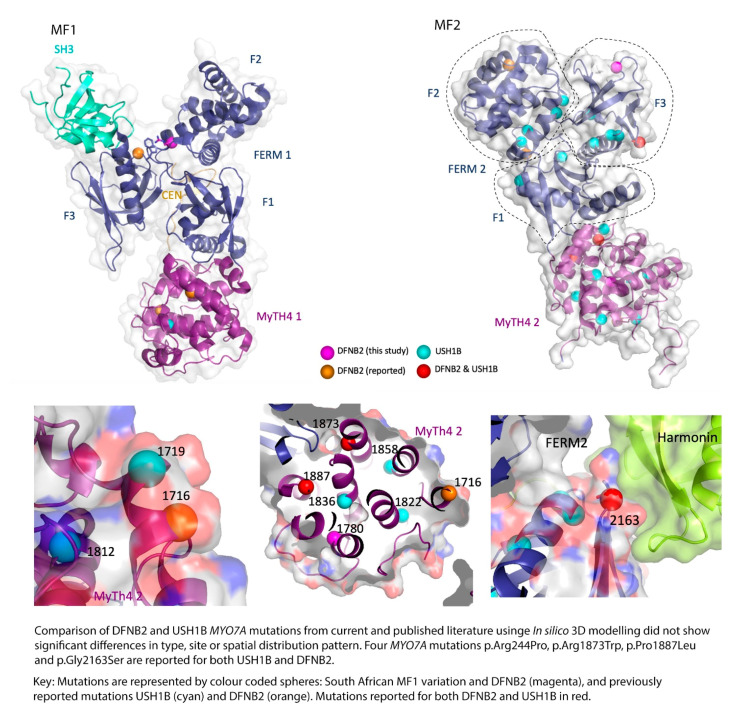
In silico models comparing location of DFNB2 and USH1 mutations.

Looking at the reported myosin VIIA mutations in published literature and the affected families, and comparing them to the results of the current study, there does not seem to be much difference in the type, site, or spatial distribution pattern of the mutations causing DFNB2 in comparison to USH1B (Table 3, Figure 4 and Figure 5). The DFNB2 mutations identified in the current study in the MyTH4-FERM domains fall into class 1 or class 2 according to the above classification. These mutations are either in the core or partial solvent exposed (Figure 4 and Figure 5). We further mapped the reported USH1B and DFNB2 mutations from published literature to investigate whether there was any significant difference in spatial distribution between the phenotypes and found overlapping results (Figure 4 and Figure 5). Both characteristics were demonstrated for mutations causing DFNB2 and USH1B. Of note, four identical mutations are reported for both USH1B and DFNB2 in published literature. These are p.Arg244Pro, p.Arg1873Trp, p.Pro1887Leu and p.Gly2163Ser (Table 3). Although we did not specifically search for it in the current study, we are yet to identify any possible vision-sparing factor in the South African DFNB2 cohort. We can only conclude that there may well be a modifying factor or gene that influences the phenotypic outcome.

We also identified an interesting heterozygous variation, Arg1463His, which falls in the highly conserved junction of the FERM1 clover leaf structure, in a South African deaf proband (Table 1, Figure 2e). It is close to the previously reported DFNB2 mutation, p.Thr1496Lys, and the USH1B mutations p.Ala1492Val and p.Leu1484Phe. The cloverleaf configuration is known to control not only the three lobes of the FERM domain but the rotational freedom of the MyTH4 as well. Mutations at this site would be predicted to cause disruption of the protein function and lead to deafness if identified in the homozygous or compound heterozygous states. This particular South African proband, however, did not demonstrate a second variation to warrant classification as DFNB2. We will watch out for this variation in future studies in the population.

## 5. Conclusions

The search for prevalent deafness genes among sub-Saharan African populations has been long and fraught with challenges due to the extreme heterogeneity of hearing loss and the tendency of population-based clustering of deafness genes [95]. We have now identified *MYO7A* as a key deafness gene among the indigenous sub-Saharan African populations from the Limpopo Province of South Africa. This was made possible by the use of Next Generation Sequencing technologies. Using a 180 gene panel, capture targeted massively parallel sequencing yielded *MYO7A* variations in 10 out of 94 probands, resulting in the identification of nine DFNB2 families in the study population, a relatively high yield that underlines the importance of *MYO7A* in this African population.

When DFNB2 was proposed as a distinct form of NSHL in 1997, families were sought to confirm the phenotype. Most reported families have come from populations of Middle Eastern, Chinese and North African descent. We have added nine families to the published 14 DFNB2 families worldwide. This is the first report of DNFB2 in sub-Saharan African families. With perfect cosegregation, the spectrum of *MYO7A* mutations in this South African population confirms DFNB2 as a specific entity that may occur in a homozygous or in a compound heterozygous state.

Although some of the families had reported consanguinity, this is not a widespread cultural practice. In fact, the practice is widely discouraged among the sub-Saharan African populations. The cause of the recurring mutations, especially Pro1780Ser, in this cohort could be due to a founder effect, but allele estimates would first have to be made to confirm this.

Our study did not identify a factor, be it a type of mutation or domain or subdomain location of mutation, responsible for the preservation of vision among DFNB2 individuals when compared to USH1B individuals. We believe a modifying gene is the likely reason. Further studies are scheduled to look into this.

## Figures and Tables

**Table 1:** Summary of genotypes in the sub-Saharan South African DFNB2 families.

cDNA	HGVS Protein Notation	Sequence_Ontology	GRCh38.p12	Family IDs	Cosegregation in Family	Novelty ClinVar	ClinVar Allele ID (Accession)	ACMG Criteria with HL-EP Specifications
c.1849T>C	p.Ser617Pro	missense	chr11:77172799	Family B BS044	Yes	ClinVar Uncertain significance (26 August 2019)	431763 (VCV000438172)	Pathogenic PP1_Strong PM3_Very Strong
c.986G>A	p.Gly329Asp	missense	chr11:77158413	Family D1 TS074/TS093	Yes	Novel	Not reported	Pathogenic PP1_Strong, PM3_Very Strong
c.5339A>C	p.Tyr1780Ser	missense	chr11:77204088	Family A TS065/TS100	Yes	ClinVar Uncertain significance (10 Febuary 2017)	511947 (VCV000521209)	Pathogenic PP1_Strong, PM3_Very Strong PS3_Supporting
				Family D2	Yes			
				Family D3	Yes			
				Family D1 TS074/TS093	Yes			
				Family C TS076	Yes			
c.6375delC	p.Pro2126Leufs*5	frameshift	chr11:77,212,972	Family C TS076	Yes	Novel	Not reported	Pathogenic PVS1_Strong PP1_Moderate PM3_Very Strong PS3_Supporting
				TS040	N/A Family not available			
c.4388G>A	p.Arg1463His	missense	chr11:77197545	TS081	N/A Family not available	Novel	Not reported	Likely Pathogenic PM2
c.1118G>A	p.Arg373His	missense	chr11:77160200	TS036	N/A Family not available	Novel	Not reported	Likely Pathogenic PM3_Supporting
c.1142C>T	p.Thr381Met	missense	chr11:77160224	TS036	N/A Family not available	ClinVar Uncertain significance (30 Junuary 2017)	546742 (VCV000552693)	Likely Pathogenic PM3_Supporting
c.1554+7C>T		splice region	chr11:77162337	TS040	N/A Family not available	ClinVar Conflicting interpretations pathogenicity (31 December 2019)	178242 (VCV000178480)	Likely Pathogenic PS1, PM3_Moderate
c.247C>A	p.Arg83Cys	missense	chr11:77147912	TS070	N/A Family not available	ClinVar Uncertain significance (30 August 2018)	552016 VCV000560896	Likely Pathogenic PP1, PM3_Supporting.

**Table 2:** Reported DFNB2 families with characterized *MYO7A* mutations.

Race/Population Group	Number of DFNB2 Families in Cohort	Citation	Summary of Families	cDNA	Protein Change
Indigenous sub-Saharan South Africans	9/89	Current study	Nine families identified with either homozygous or compound heterozygous mutations in *MYO7A* leading to DFNB2	c.247C>A	p.Arg83Cys
				c.986G>A	p.Gly329Asp
				c.1118G>A	p.Arg373His
				c.1142C>T	p.Thr381Met
				c.1849T>C	p.Ser617Pro
				c.5339A>C	p.Tyr1780Ser
				c.6375delC	p.Pro2126Leufs*5
Iranian	2/30	Asgharzade et al., 2017	Two out of 30 deaf families displayed linkage to and were cofirmed DFNB2	c.6487G>A	p.G2163S
				c.448 C>T	p.Arg150X
Moroccan	2/61	Bakhchane A et al., 2017	Compound heterozygous mutations	c.6025delG	p.Ala2009Profs*32
				c.6229T>A	p.Trp2077Arg
				c.3500T>A	p.Leu1167His
				c.5617C>T	p.Arg1873Trp
				c.4487C>A	p.Thr1496Lys
Iraqi	1/1	Ben-Salem et al., 2014	Homozygous mutations	c.1952_1953insAG	p.Cys652Glyfs*11
Palestinians	1/1	Ben-Salem et al., 2014	Homozygous mutations	c.5660C>T	p.Pro1887Leu
Pakistani	1/24	Riazuddin et al., 2008	Segregated nonsyndromic hearing loss due to a novel three-nucleotide deletion in an exon of MYO7A encoding a region of the tail domain	c.5142_5144del	p.Glu1716del
Chinese	2/2	Liu et al., 1997	Homozygous and compound heterozygous mutations	c.133-2A>G	Splice region p.Arg244Pro
				c.731G>C	p.V1199insT
Jewish	2/248	Brownstein et al., 2014	Compound heterozygous and homozygous mutations	c.29T4C	p.Val10Ala
				c.1969C4T	p.Arg657Trp
				c.620A4G	p.Asn207Ser
Arab Palestinian	3/611	Brownstein et al., 2014	Homozygous mutations (including splice site mutations)	c.4153-2A4G	Splice region
				c.6211C4T	p.Gln2071*
				c.G6487A	p.Gly2163Ser

**Table 3:** Spectrum of *MYO7A* mutations reported on ClinVar among DFNB2 and USH1B families.

Phenotype	Nucleotide Change	Protein Change	Citation	Ref
DFNB2	c.29T>C	p.Val10Ala	Brownstein et al., 2014	[54]
DFNB2	c.247C>A	p.Arg83Cys	Current study	
DFNB2	c.133-2A>G	Splice acceptor variant	Liu et al., 1997	[50]
DFNB2	c.448C>T	p.Arg150Ter	Asgharzade et al., 2017	[58]
DFNB2	c.620>4G	p.Asn207Ser	Brownstein et al. 2014	[54]
DFNB2	c.731G>C	p.Arg244Pro	Liu et al., 1997	[50]
DFNB2	c.986G>A	p.Gly329Asp	Current study	
DFNB2	c.1118G>A	p.Arg373His	Current study	
DFNB2	c.1142C>T	p.Thr381Met	Current study	
DFNB2	c.1554+7C>T	Splice region	Current study	
DFNB2	c.1849T>C	p.Ser617Pro	Current study	
DFNB2	c.1969C>T	p.Arg657Trp	Brownstein et al., 2014	[54]
DFNB2	c.1952_1953insAG	p.Cys652Glyfs*11	Ben-Salem et al., 2014	[57]
DFNB2	c.3500T>A	p.Leu1167His	Bakhchane A et al., 2017	[61]
DFNB2	c.4153-2A>G	Splice acceptor variant	Brownstein et al., 2014	[54]
DFNB2	c.4388G>A	p.Arg1463His	Current study	
DFNB2	c.4487C>A	p.Thr1496Lys	Bakhchane A et al., 2017	[61]
DFNB2	c.5146_5148delGAG	p.Glu1716del	Riazuddin et al., 2008	[19]
DFNB2	c.5339A>C	p.Tyr1780Ser	Current study	
DFNB2	c.5617C>T	p.Arg1873Trp	Bakhchane A et al., 2017	[61]
DFNB2	c.5660C>T	p.Pro1887Leu	Ben-Salem et al., 2014	[54]
DFNB2	c.6025delG	p.Ala2009Profs*32	Bakhchane A et al., 2017	[61]
DFNB2	c.6211C>T	p.Gln2071*	Brownstein et al., 2014	[54]
DFNB2	c.6229T>A	p.Trp2077Arg	Bakhchane A et al., 2017	[61]
DFNB2	c.6375delC	p.Pro2126Leufs*5	Current study	
DFNB2	c.G6487A	p.Gly2163Ser	Ammar-Khodja et al., 2009	[71]
USH1B	c.252C4G	p.Asn84Lys	Riazuddin et al., 2008	[19]
USH1B	c.398_399insC	p.His133fs*139	Riazuddin et al., 2008	[19]
USH1B	c.495delG	p.Glu166fs*170	Riazuddin et al., 2008	[19]
USH1B	c.640G>A	p.Gly214Arg	Adato et al., 1997	[72]
USH1B	c.731G>A	p.Arg244Pro	Liu et al., 1997	[50]
USH1B	c.471-1G>A	Splice acceptor variation	Riazuddin et al., 2008	[19]
USH1B	c.977T>A	p.Leu326Gln	Riazuddin et al., 2008	[19]
USH1B	c.1309G4A	p.Asp437Asn	Riazuddin et al., 2008	[19]
USH1B	c.1591C4T	p.Gln531X	Riazuddin et al., 2008	[19]
USH1B	c.1935+1G>A	Splice region	Riazuddin et al., 2008	[19]
USH1B	c.2476G>A	p.Ala826Thr	Adato et al., 1997	[72]
USH1B	c.2914C4T	p.Arg972X	Riazuddin et al., 2008	[19]
USH1B	c.3135_3136insC	p.Leu1046fs*1054	Riazuddin et al., 2008	[19]
USH1B	c.3134T>C	p.Ile1045Thr	Jaijo et al., 2007	[73]
USH1B	c.3502C>T	p.Arg1168Pro/Trp	Le Guédard-Méreuze et al., 2010	[74]
USH1B	c.3508G>A	p.Glu1170Lys	Cuevas et al., 1999	[75]
USH1B	c.3631delT	p.Tyr1211fs*1231	Riazuddin et al., 2008	[19]
USH1B	c.3652G>A	p.Gly1218Arg	Duzkale et al., 2013	[76]
USH1B	c.3719G>A	p.Arg1240Gln	Janecke et al., 1999	[77]
	c.3718C>T	p.Arg1240Trp	Vaché et al., 2010	[78]
USH1B	c.3979G>A	p.Glu1327Lys	Nájera et al., 2002	[79]
USH1B	c.4029G>C	p. Arg1343Ser	Janecke et al., 1999	[77]
USH1B	c.4450C>T	p.Leu1484Phe	Jaijo et al., 2006	[80]
USH1B	c.4697C>T	p.Thr1566Met	Nájera et al., 2002	[79]
USH1B	c.4805G>A	p. Arg1602Gln	Liu et al., 1998	[43]
USH1B	c.4838delA	p.Asp1613fs*1644	Riazuddin et al., 2008	[19]
USH1B	c.5146_5148delGAG	p.Glu1716del	Riazuddin et al., 2008	[19]
USH1B	c.5156A>G	p.Tyr1719Cys	Janecke et al., 1999; Cuevas et al., 1999	[75,77]
USH1B	c.5366+1G>A	Splice region	Riazuddin et al., 2008	[19]
USH1B	c.5434G>A	p.Glu1812Lys	Roux et al., 2011	[81]
USH1B	c.5464A>C	p.Thr1822Pro	Duzkale et al., 2013	[76]
USH1B	c.5507T>C	p.Leu1836Pro	Jaijo et al., 2007	[73]
USH1B	c.5573T>C	p.Leu1858Pro	Bharadwaj et al., 2000	[82]
USH1B	c.5618G>A	p.Arg1873 Gln	Cremers et al., 2007	[83]
	c.5617C>T	p.Arg1873 Trp	Roux et al., 2006	[84]
USH1B	c.5648G>A	p.Arg1883Gln	Ouyang et al., 2005	[85]
USH1B	c.5804T>C	p.Leu1935Pro	Duzkale et al., 2013	[76]
USH1B	c.5824G>A	p.Gly1942Arg	Duman et al., 2011	[86]
USH1B	c.5944G>A	p.Gly1982Arg	Riazuddin et al., 2008	[19]
USH1B	c.6028G>A	p.Asp2010Asn	Chen et al., 2016	[87]
USH1B	c.6062A>G	p.Lys2021Arg	Roux et al., 2011	[81]
USH1B	c.6326C>T	p.Thr2109Ile	Duzkale et al., 2013.	[76]
USH1B	c.6487G>A	p.Gly2163Ser	Janecke et al., 1999	[77]
USH1B	c.6560G>A	p.Gly2187Asp	Bharadwaj et al., 2000	[82]
USH1B	c.6557T>C	p.Leu2186Pro	Bonnet et al., 2011	[88]
USH1B	c.6577C>	p.Leu2193Phe	Duzkale et al., 2013	[76]

Amino acid positions in red represent positions reported for both DFNB2 and USH1B.

## Data Availability

Data is contained within the article and supplementary material, as well as in our previously published papers [69,70,95].

## Supplementary Materials

The following are available online at https://www.mdpi.com/2073-4425/12/2/274/s1, Table S1: Summary of MYO7A allele and genotype distribution in the sub-Saharan South African DFNB2 families, Table S2: Alignment of the conserved second MyTH7 subdomain in different species and against p.Pro2126Leufs*5, Figure S1: Sanger sequencing electropherograms of *MYO7A* mutations among South African DFNB2 families.
