# Supplementary material for: Spectrum of MYO7A Mutations in an Indigenous South African Population Further Elucidates the Nonsyndromic Autosomal Recessive Phenotype of DFNB2 to Include Both Homozygous and Compound Heterozygous Mutations
Source: Genes (Basel). 2021 Feb 15;12(2):274. doi: 10.3390/genes12020274 (PMC7919343; doi:10.3390/genes12020274)
Supplement: Supplementary file 1 [file genes-12-00274-s001.pdf]

Table S1. Summary of MYO7A allele and genotype distribution in the sub-Saharan South African DFNB2 families
Available family members_DNA ∑N = 32   Total number of individuals affected_Deaf   ∑n = 17   Total number of variations = 8

MYO7A variation	Reference allele	Genotype	Family 1 TS065/100 *n* = 5	Family 2 TS074/093 *n* = 5	Family 3 BS044 *n* = 9	Family 4 TS076 *n* = 5	Family 5 Shilo (TS074 branch C) *n* = 3	Family 6 (TS074 branch B) *n* = 2	Family 7 TS040 *n* = 1*	Family 8 TS036 *n* = 1*	Family 9 TS070 *n* = 1*
p.Tyr1780Ser	A	C/C	2				2	1			
		A/C	3	4		3	1	1			
p.Pro2126LeuTer5	TC	TC/T				5			1		
splice_ region									1		
p.Ser617Pro	T	C/C			3						
		T/C			4						
p.Gly329Asp	G	G/A		4							
p.Arg373His	G	G/A								1	
p.Thr381Met	C	C/T								1	
p.Arg83Cys	C										1
**Total affected (n)**			**2**	**3**	**3**	**3**	**2**	**1**	**1**	**1**	**1**

Table S2. Alignment of the conserved second MyTH7 subdomain in different species and against p.Pro2126Leufs*5.

```
WT              CAA ACT ACG GAG CCA AAC TTC CCT GAG ATC CTC CTA ATT GCC ATC AAC AAG TAT GGG GTC AGC CTC ATC GAT CCC AAA
Translation +1   Q   T   T   E   P   N   F   P   E   I   L   L   I   A   I   N   K   Y   G   V   S   L   I   A   P   L
Family TS076 MT CAA ACT ACG GAG CCA AAC TTC CTG AGA TCC TCC TAA
                 Q   T   T   E   P   N   F   L   R   S   S   *
```

2114 FFEVKQTTEPNFLR S S* p.Pro2126Leufs*5
HS mutant

2114 FFEVKQTTEPNFPE I LLIAINKYGVSLID PKTKDILT THPFTKISNWSSGNTYFHI TIGNLVRGSKLLCETSLGYKMDDLLTSYISQMLTAM
HS Wild type
2068 FFEVKQTTEPNYPE M LLIAINKHGVSLIH PVTKDILV THPFTRISNWSSGNTYFHMTIGNLVRGSKLLCETSLGYKMDDLLTSYISL MLTNM
DM
2064 FFEVKQTTEPNYPE M LLIAINKHGVSLIH PSSKDILV THPFTRISNWSSGNTYFHM TIGNLVRGSKLLCETSLGYKMDDLLTSYISL MLTNM
AA
2076 FFEVKQTTEPHFPE I LLIAINKYGVSLID PKNKDILT TYPFTKISNWSSGNTYFHI TIGNLVQGSKLLCETSLGYKMDDLLTSYISQMLTTM
DR
2114 FFEVKQTTEPNFPE I LLIAINKYGVSLID PRTKDILT THPFTKISNWSSGNTYFHI TIGNLVRGSKLLCETSLGYKMDDLLTSYISQMLTAM
MM
2076 FFEVKQTTEPNFPE I LLIAINKYGVSLID PRTKDILT THPFTKISNWSSGNTYFHI TIGNLVRGSKLLCETSLGYKMDDLLTSYISQMLTAM
SS
2068 FFEVKQTTEANYAE M LLIAINKHGVSLIH PVTKDILV THPFTRISNWSSGNTYFHMTIGNLVRKLLCETSLGYKMDDLLTSYISL MLTNM
DM mutant

V1 V2

HS *Homo sapiens;* **DM** *Drosophila melanogaster*; **AA** *Anopheles arabienis*; **DR** *Danio rerio*; **MM** *Mus musculus*; **SS** Sus scrofa

Mutation p.Pro2126Leufs*5 results in a frameshift with termination of the protein after residue 5 downstream. The premature termination of the protein leads to interference with the conformation and regulation of the motor function of the motor domain. In their functional studies, Yang et al (2009) demonstrated that a point mutation induced at V2 abolished the folding of the tail region over the head domain and the autoregulation of the myosin 7a protein. *Adapted from Yang et al., 2009.*

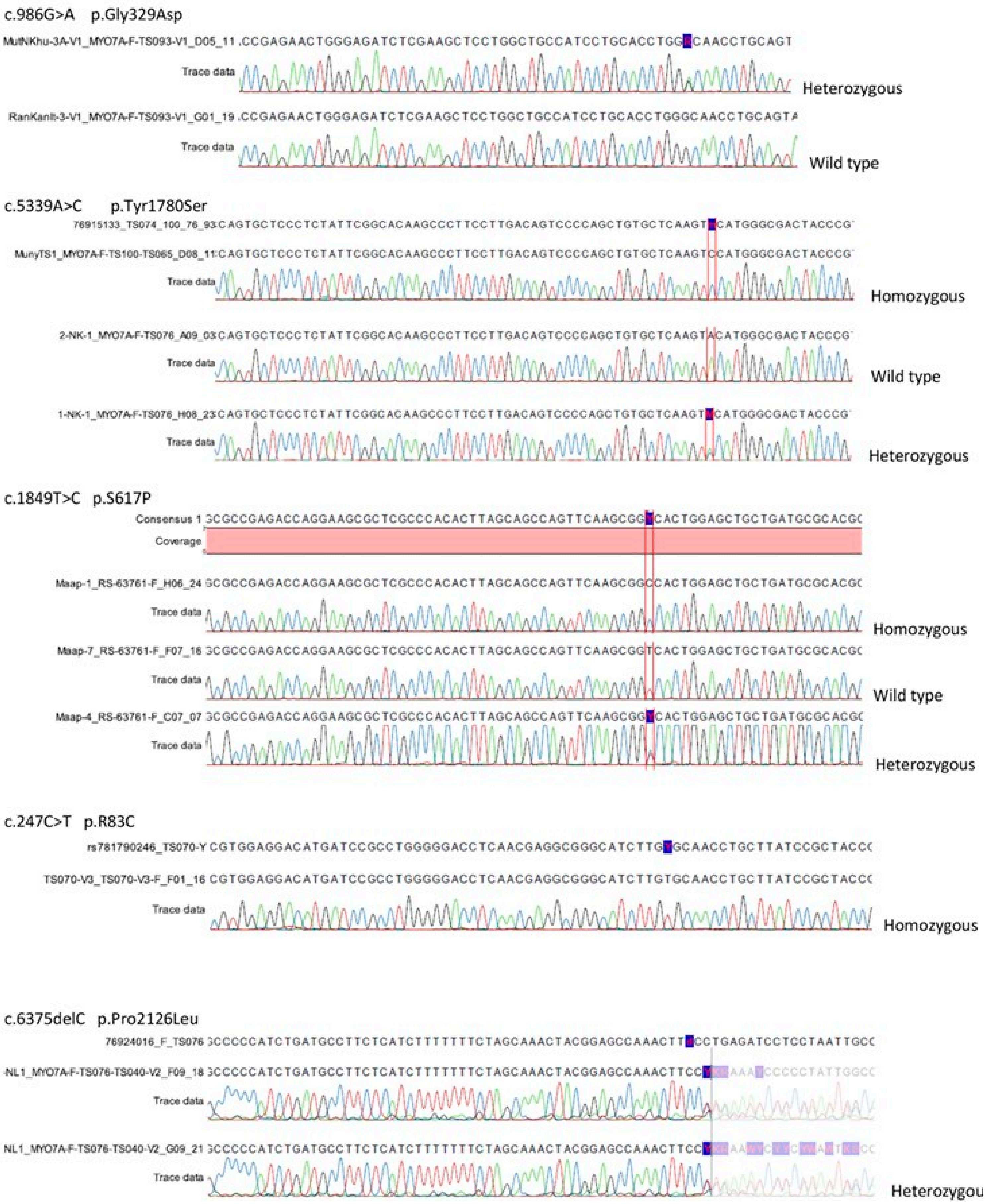

Figure S1: Sanger sequencing electropherograms of *MYO7A* mutations among South African DFNB2 families.
